# Addressing obstetric violence: a scoping review of interventions in healthcare and their impact on maternal care quality

**DOI:** 10.3389/fpubh.2024.1388858

**Published:** 2024-06-24

**Authors:** Abena Asefuaba Yalley, Gabija Jarašiūnaitė-Fedosejeva, Burcu Kömürcü-Akik, Liliana de Abreu

**Affiliations:** ^1^Zukunftskolleg, University of Konstanz, Konstanz, Germany; ^2^Department of Politics and Public Administration, University of Konstanz, Konstanz, Germany; ^3^Department of Psychology, Vytautas Magnus University, Kaunas, Lithuania; ^4^Department of Psychology, Ankara University, Ankara, Türkiye

**Keywords:** obstetric violence, humanized childbirth, facility-based delivery, interventions, women

## Abstract

**Background:**

The mistreatment and abuse of women during childbirth have been recognized as a major global health challenge, impeding facility-based delivery and contributing to the high maternal mortalities globally. The World Health Organization has specifically called for interventions to deal with obstetric violence. This scoping review consolidates the existing literature on interventions aimed at reducing obstetric violence and synthesizes existing knowledge on their impact in promoting respectful maternity care.

**Methodology:**

Thirteen electronic databases were searched for relevant articles from January 2001 to March 2023. A total of 863 records were identified, and 72 full-text articles were retrieved for further screening. The review includes 16 studies, particularly from low- and middle-income countries, with interventions implemented at medical facilities and involving both women and healthcare providers. Eight of the studies were quantitative, three were qualitative and five used a mixed-methods approach.

**Findings:**

The results reveal a promising trend in reducing obstetric violence through various interventions. Ten different types of interventions were identified, highlighting strategies to improve the quality of maternity care and enhance patient-centered care. Improved patient-provider communication skills, increased privacy measures, and reduced abuse and mistreatment emerged as common themes. Enhanced communication skills, including open discussions and the right to be informed, were crucial in reducing obstetric violence. Privacy measures, such as separate rooms, curtains, and birth companions effectively decreased incidents of non-confidential care. General abuse and mistreatment, including physical abuse and neglect, were also reduced, leading to improved perceptions of respectful care during childbirth.

**Conclusion:**

Overall, the interventions had a favorable impact on obstetric violence reduction and women’s childbirth experiences. However, despite promising results, obstetric violence remains prevalent worldwide, necessitating more efforts to implement effective interventions. To the best of our knowledge, this is the first scoping review on obstetric violence interventions, providing a comprehensive overview of the state of the art. We suggest that further research is needed to explore new interventions, particularly gender-sensitive interventions, to contribute to a growing body of knowledge on the prevention of obstetric violence.

## Introduction

The Charter on the Universal Rights of Childbearing Women emphasizes the fundamental right to receive dignified and respectful sexual and reproductive healthcare, including during childbirth (1). Thus, mistreatment and violence during childbirth are considered a violation of women’s fundamental human rights. Worldwide, there is clear evidence that demonstrates that a substantial number of women experience poor standards of care and mistreatment during childbirth. These mistreatments, widely conceptualized as obstetric violence, have been recognized as a major global health challenge with devastating impacts on women’s and children’s health. According to Vacaflor (2), obstetric violence refers to ‘the violence exercised by health personnel on the body and reproductive processes of women (during pregnancy or childbirth), as expressed through dehumanizing treatment, medicalization abuse, and the conversion of natural processes of reproduction into pathological ones. Obstetric violence as a concept is relatively new in the global health literature with researchers adopting various terminologies such as ‘disrespect and abuse (D&A),’ ‘mistreatment and abuse,’ ‘dehumanized childbirth,’ and ‘disrespectful maternity care’ to describe violence and abuse in obstetric care. While these terminologies help to clearly categorize the different manifestations of violence, the term ‘obstetric violence’ emphasizes the ‘structural dimensions as a gender-based violence that intersects with institutional violence’ (3, 4). Nonetheless, all the terminologies acknowledge the harmful effects of violence, the dehumanization of childbirth and the violation of women’s rights and dignity. In this study, we use the term ‘obstetric violence’ interchangeably with the other terminologies to cover vast literature. Obstetric violence could manifest in the form of physical violence, denial of birth companion, intimidation, forced medical care, neglect or abandonment, lack of confidentiality, failure to seek consent, unjustified cesarean sections, restrictions on food and mobility, and excessive use of oxytocin to induce labor (3, 5–7). Women are often stripped of the agency to make decisions over their bodies, while some are subjected to dehumanizing treatments. The World Health Organization has recognized it as a torturous phenomenon that is very widespread and called for critical studies that interrogate obstetric violence and interventions to reduce it.

Mistreatment of women during childbirth has been recorded in both high-income (8, 9) and middle-to low-income countries (10–12). Recent studies on obstetric violence reveal a prevalence of 33% in Mexico, 44% in Argentina, 76% in Türkiye, 15% in India, and 17% in the United States (13–17). In Africa, some obstetric violence rates have also been reported: 20% in Kenya, 20–28% in Tanzania, 65.3% in Ghana, 78% in Ethiopia, and 98% in Nigeria (18–22). Similarly, a multi-country study conducted in four Sub-Saharan African countries found that 40% of women experienced obstetric violence during facility birthing (18). Research has also demonstrated that obstetric violence is associated with a higher risk of maternal problems such as obstructed labor and postpartum hemorrhage and even death (23). The psychological consequences of obstetric violence following childbirth include depression and post-traumatic stress disorder (PTSD) (24). The (anticipated) experience of obstetric violence prevents many women from utilizing facility-based services for childbirth (3). Violence during childbirth is the most cited reason for women’s refusal to reuse health facilities in subsequent pregnancies in Latin America (25). As a consequence, this increases women’s risks of preventable complications and maternal mortality. Hence, reducing maternal mortality requires the strengthening of institutional health, particularly of maternity care systems, and addressing barriers that limit access and reduce the quality of obstetric services.

While previous studies have made important contributions to the discourse in this field, the explicit focus on interventions to reduce the prevalence of obstetric violence has not been well articulated in the literature. Particularly, consolidated literature that documents existing interventions for obstetric violence and their effectiveness in reducing the occurrence of violence during obstetric care is scarce. Therefore, the present study reviews the available literature on obstetric violence interventions to examine existing interventions and their effectiveness. We explore the different types of interventions, their underlying theories of change, and their impact on patient knowledge, provider attitudes, communication, and overall quality of care. By examining these interventions, this review aims to provide a comprehensive overview of the strategies used to promote respectful and patient-centered maternity care.

The term ‘intervention’ is used in this study to refer to any actions, programs, or activities that are implemented with the primary aim of improving human health and achieving a better health outcome (26). In the context of obstetric violence, interventions can include programs, activities and actions aimed at reducing violence during childbirth. A review that explores the diverse programs, approaches or interventions builds an evidence base that illuminates the range of interventions used to address this critical global health challenge. This is an important contribution to a growing body of evidence in this field and one that seeks to improve reproductive health and women’s rights within the overall childbearing discourse. This review provides timely input to those seeking to design impactful interventions to address the problem of violence during childbirth to improve the quality of maternity care around the world.

## Materials and methods

### Inclusion and exclusion

This scoping review examined obstetric violence interventions and synthesized the impact of the interventions in dealing with abuse during childbirth. The study particularly examined a range of interventions implemented to reduce abuse and disrespect during childbirth, enhance patient-provider interactions, and improve the overall quality of obstetric care. We included all primary research (qualitative and quantitative) aimed at evaluating the impact of obstetric violence interventions and only academic peer-reviewed articles published in English. Due to the contemporary nature of the emergence of obstetric violence research, the timeline for the search was from January 2000 to March 2023. For inclusion, the study must have described the interventions that were implemented and utilized empirical data to measure post-intervention outcomes. The interventions must solely focus on dealing with obstetric violence. Studies that described obstetric violence interventions but did not implement the interventions nor evaluate the outcome were excluded. Furthermore, this study excluded publications that were merely based on secondary materials such as literature reviews, commentaries, systematic reviews and descriptions of policies and laws as well as study protocols.

### Outcome of interest

The outcome of interest in this scoping review includes a comprehensive understanding of the impact and effectiveness of interventions targeted at reducing obstetric violence. The chosen outcomes align with Bowser and Hills’ (27) framework, which categorizes obstetric violence into distinct forms, allowing for a broader exploration of the interventions. Therefore, the key outcomes included addressing physical abuse, ensuring informed consent for medical procedures, preserving confidentiality, promoting dignified care, eliminating discrimination, and mitigating instances of abandonment or detention during childbirth. Studies included must have measured at least one of these outcomes. These outcomes collectively provide a deeper understanding of the effectiveness of interventions across various dimensions of obstetric violence, contributing valuable insights to the broader discourse on improving maternity care and fostering respectful practices worldwide.

### Literature search

The review followed the four principal steps according to The Preferred Reporting Items for Systematic Reviews and Meta-Analyses (PRISMA) framework by Liberati et al. (28). These include identification, screening, eligibility, and inclusion. A systematic search was carried out on the Cumulative Index to Nursing and Allied Health Literature (CINAHL), EBSCOhost, PubMed, Web of Science, African online journals, Cochrane Library, SciVerse, Scopus, Google Scholar, PsychINFO, PsychArticles, Medical Literature Analysis and Retrieval System Online, and Maternity and Infant Care to retrieve relevant literature in November 2022. The search terms broadly combined using Boolean expressions formed the first stage of the searching and screening for this review. The search terms and their combinations (see Table 1) were adapted to the specifics of each electronic database.

**Table 1:** Complete list of keywords for search.

Concept (obstetric violence)	“Obstetric violence” OR “abuse during childbirth” OR “disrespect and abuse during childbirth” OR “dehumanization of childbirth” OR “dehumanized care” OR “childbirth” OR “disrespect and abuse” OR “mistreatment during childbirth” OR “disrespectful maternity care” OR “respectful maternity care” OR “mistreatment,” OR “neonatal care” OR “humanized care” OR “institutional violence”
Interventions	AND “interventions” OR “strategies” OR “best practice” OR “response” OR “reduce” OR “prevent” OR “support” “address”
Populations	AND “women” OR “healthcare professionals” OR “postpartum women” OR “pregnant women”
Time	2001–2022

### Search strategy

**Figure 1:**
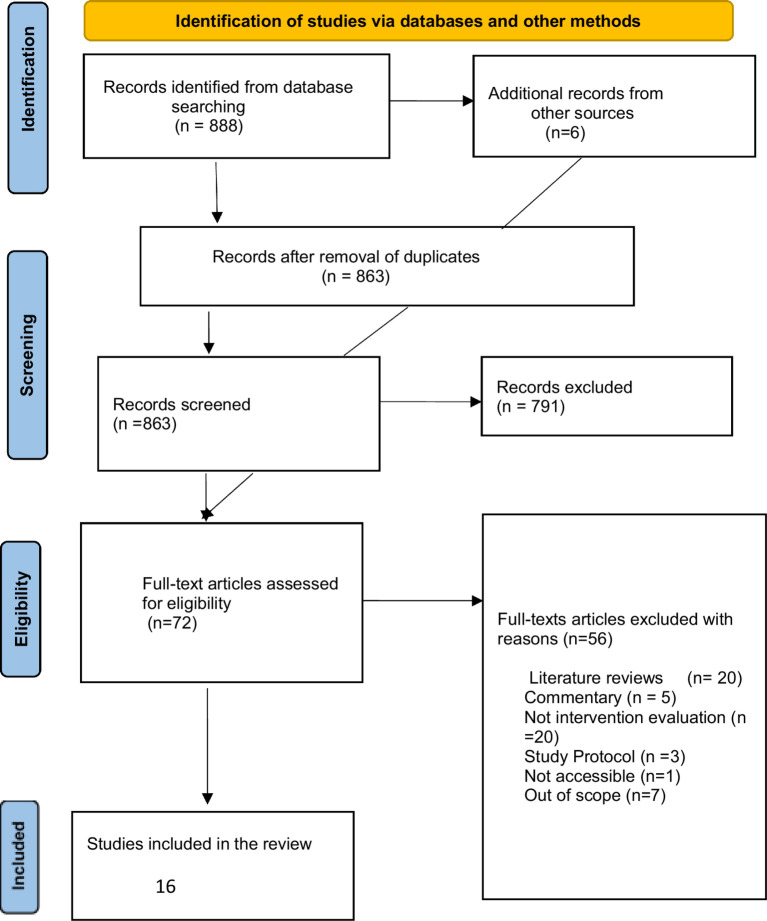
PRISMA flow chart.

The search generated a total of 888 items for screening in the first mapping phase. The results were combined with references suggested by experts and through bibliographic searches, which yielded six additional studies, making a total of 894 studies. A second search was conducted in March 2023 to include studies published from December 2022 to March 2023, but no new study was identified. During the screening, 31 duplicates were identified and deleted. Items identified in the search were then screened for inclusion in the mapping, initially based on title and abstract. The team screened the full text when inclusion or exclusion could not be determined from the title and abstract. The subsequent screening involved two team members (AAY, GJF) who independently screened the 72 items, compared results and resolved any differences in understanding the inclusion/exclusion criteria. The other team members (LA and BKA) were involved when AAY and GJF could not agree on any full-text item. In such cases, decisions were made in favor of an inclusive approach. After a thorough screening of the papers, a total of 16 items were included in the mapping. Figure 1 shows the flow diagram of the searching and screening strategy.

### Data extraction and analysis procedure

The following data were extracted for each selected study: name of the first author, year publication, country where the study was undertaken, information about the study (article type, design and aims), study population (sample size and targeted population), type of intervention, length of intervention, control group, data collection mode, measurement point, data analysis, outcome measures and a concise description of the findings of the study. Significant evidence relevant to the goal of the review was systematically extracted, appraised, and reported using the PRISMA-ScR checklist (28, 29). The outcomes of the extracted data were synthesized to answer the research question.

## Results

### General characteristics of the studies

#### Study design

Overall, 16 articles were included in the study, with the earliest study published in 2001 while the most recent studies were published in 2021. Thirteen of the reviewed studies employed a comparative before-and-after intervention evaluation approach (19, 30–41). Eight studies used quantitative data (19, 30, 31, 35–38, 40), five studies used a mixed-methods approach (32–34, 39, 41) and three studies collected qualitative data (42–44). The 16 studies were geographically diverse, conducted in 9 countries: Tanzania (*n* = 2), Kenya (*n* = 1), Ethiopia (*n* = 3), Ghana (*n* = 3), Sri Lanka (*n* = 2), Brazil (*n* = 2), South Africa (*n* = 1), Sudan (*n* = 1), and Sweden (*n* = 1). The publication timeline spanned two decades, from 2001 (39) to 2021 (31, 43). A detailed list of the studies and extracted data is presented in Table 2.

**Table 2:** Characteristics of included articles (*n* = 16).

No.	Author (Year)/country	Study 1. Article type 2. Design 3. Aims	Population 1. Total participants (N) 2. Recipient of an intervention	Methodology 1. Intervention type 2. Intervention length 3. Control group (yes/no) 4. Data collection mode 5. Measurement points 6. Data analysis 7. Outcome measures	Findings
1.	Kujawski et al., (2017)/Tanzania	1. Quantitative study. 2. Comparative before-and-after evaluation design. 3. To test interventions to reduce disrespectful and abusive treatment of women during labor and delivery.	1. 1,388 women at baseline and 1,680 women at endline. 2. Community and health system stakeholders, and hospital staff.	1. The Staha intervention. 2. 27 months (the charter process took over 6 months + the study team assisted in the facilitation of the quality improved process for 11 months and intervention managed independently by facility managers for 10 months). 3. Yes. 4. Exit interview 5. At baseline and 10 months after support for the intervention’s implementation ended. 6. Multivariable logistic regression to estimate a difference-in-difference model; generalized linear models with a Poisson distribution. 7. Experience of disrespectful or abusive actions during labor and delivery; the association between the intervention and delivery satisfaction and quality of care.	66% reduced odds of women experiencing disrespect and abuse during childbirth. The biggest reductions were for physical abuse and neglect.
2.	Mengistu et al., (2021)/Ethiopia	1. Quantitative study. 2. Comparison of changes in routinely (monthly) collected data. 3. To describe the development, implementation, and results of intervention to improve respectful maternity care.	1. 107 healthcare providers from 17 health centers and 3 hospitals. 2. Health care providers from three districts in Ethiopia (multidisciplinary health professionals, including facility leadership, MNH clinical providers, data managers and health extension workers)	1. Respectful maternity care training module with three core components: testimonial videos developed from key themes identified by staff as experiences of mothers, skills-building sessions on communication, and onsite coaching. 2. 11 months. 3. No. 4. Senior project officers at the Institute for Health Care Improvement collected the data and entered these into a program database as part of their routine work. 5. Monthly data collection. 6. Interrupted time series and regression analysis. 7. Facility-level solutions applied to enhance the experience of care were documented. Safe Childbirth Checklist data measuring privacy and birth companion offered during labor and childbirth were collected over 27 months from 17 health centers and 3 hospitals.	Significant improvement in the percentage of births with two elements of respectful maternal care – privacy and birth companionship offered- was noted in one district, while in the other two districts, results were mixed.
3.	Mihret et al., (2020)/Ethiopia	1. Mixed methods study 2. Comparative before-and-after evaluation design. 3. To reduce the disrespect and abuse of mothers during antenatal care and delivery services at Injibara General Hospital, Northwest Ethiopia.	1. 133 healthcare providers and 748 randomly selected mothers (369 pre-intervention and 369 post-intervention). 2. Health care providers and supervisors (midwives, case managers, coordinators, porters, medical record unit coordinators, and liaison officers).	1. Provision of training, preparation of standard written guidelines and protocols, waiting room construction, availing screening or curtain, equipment, essential drugs and supplies, supportive supervision and mentoring, and staff motivation were the lists of interventions applied to decrease disrespect and abuse. 2. 6 months. 3. No. 4. Quantitative data of women were collected during an exit interview using an interviewer-administered structured questionnaire. Qualitative part data of health care providers were collected through face-to-face interviews using a semi-structured interview. 5. Pre-and-post intervention interview with women and interview with 10 informants (three health care providers, four women development army leaders, and three mothers with at least three ANC visits in the hospital) for qualitative study. 6. Descriptive statistics and independent t-test for quantitative data and thematic analysis for qualitative data. 7. Process measures (e.g., the availability of written policies, protocols, and monitoring and evaluation guidelines for improving Respectful Maternity Care practice, availability of screens or curtains, the number of supportive supervisions and mentorship conducted, etc.) and proportion of disrespect and abuse among pregnant and laboring mothers (disrespect and abuse, physical abuse, non-consented care, non-confidential care, non-dignified care, discrimination, abandonment or denial of care).	Disrespect and abuse during pregnancy and childbirth decreased from 71.8% at baseline to 15.9% at the end-line with a 55.9% change (mean difference: 0.56, 95% CI: 0.55–0.57). Alongside, the magnitude of the subscales of disrespect and abuse (physical abuse, non-consented care, non-confidential care, non-dignified care, discrimination, and neglected care) was decreased at post-intervention, compared with the baseline.
4.	Ratcliffe et al., (2016)/Tanzania	1. Mixed methods study. 2. Comparative before-and-after evaluation design. 3. To describe an exploratory study conducted in a large referral hospital in Dar es Salaam, Tanzania that sought to measure disrespect and abuse, introduce a package of interventions to reduce its incidence, and evaluate their effectiveness.	1. Baseline: 2000 women were interviewed at the hospital 3–6 h postpartum, a sub-sample of 77 women were re-interviewed in their homes 4–6 weeks after birth. 200 direct observations of interactions between women and providers during labor and delivery were conducted. 50 structured interviews (18 in-depth) with health care providers were taken as well. Post-intervention: Open Birth Days observations and 22 brief interviews with women, Pre, and Post-tests with 362 women who participated in Open Birth Days; Pre and Post-Tests with 76 healthcare professionals for Respectful Maternity care Workshop; Respectful maternity care Workshop Action Plan (monthly monitoring); direct observation of 459 women, including 57 women who attended Open Birth Days; community follow-up interviews with 149 women, including 28 women who attended Open Birth Days; structured providers interviews with 55 health care providers, including 25 who participated in the Respectful Maternity Care Workshop. 2. Women (Open Birth Days intervention) and health care providers and administrators at the study facility, district officials, and National representatives (The Respectful Maternity Care Workshop).	1. Two interventions: (1) Open Birth Days, a birth preparedness and antenatal care education program, and (2) a Respectful Maternity Care workshop for healthcare providers based on the Health Workers for Change curriculum. 2. 7 months. 3. No. 4. Pre- and post-tests with participants in Open Birth days and pre- and post-tests with participants in the Respectful Maternity Care Workshop were administered. Additionally, study staff conducted periodic observations of Open Birth Days sessions and short open-ended interviews with participants, as well as regular monitoring of progress toward achievement of the RMC Workshop action plan. 5. Pre- and-post intervention measurements. 6. Descriptive statistics. 7. Health care providers’ and women’s knowledge of patient rights, women’s knowledge of the labor and delivery process, provider and patient attitudes toward each other, provider-patient communication, women’s experiences of labor and delivery, patient satisfaction with care received, provider job satisfaction, and the quality of patient-provider relationships.	Comparisons before and after the interventions showed an increase in patient and provider knowledge of user rights across multiple dimensions, as well as women’s knowledge of the labor and delivery process. Women reported feeling better prepared for delivery and provider attitudes toward them improved, with providers reporting higher levels of empathy for the women they serve and better interpersonal relationships. Patients and providers reported improved communication, which direct observations confirmed. Additionally, women reported feeling more empowered and confident during delivery. Provider job satisfaction increased substantially from baseline levels, as did user reports of satisfaction and perceptions of care quality.
5.	Abuya et al., (2015)/Kenya	1. Quantitative study 2. Comparative before-and-after evaluation design. 3. NR	1. Baseline: 641 women and endline: 728 women. 2. Policy makers, service care providers	1. Heshima project. The intervention involved working with policymakers to encourage greater focus on disrespect and abuse, training providers on respectful maternity care, and strengthening linkages between the facility and community for accountability and governance. 2. In 6 facilities for 20 months and 7 – for 14 months. 3. No. 4. Exit interviews with women who had just delivered and observation of women, from their early labor to post-delivery, conducted by trained nurses and midwives. 5. Pre and post intervention. 6. Chi-square test, unadjusted and multivariate logistic generalized linear mixed models (GLMM), with the facility as a random effect and all other variables as fixed effects. 7. Women’s demographic and household characteristics including their socio-economic status, past service utilization, characteristics of their deliveries, their perceived quality and satisfaction, and experiences of disrespect and abuse. Three indicators for initial examination (non-consensual care, verbal abuse, lack of privacy), three during delivery (physical aggression, verbal aggression, lack of privacy), and one for postpartum care (bed sharing) were observed and documented as well.	Overall disrespect and abuse decreased from 20 to 13% (*p* < 0.004) and among four of the six typologies disrespect and abuse decreased from 40 to 50%. Night shift deliveries were associated with greater verbal and physical abuse. Patient and infant detainment declined dramatically from 8.0–0.8%, though this was partially attributable to the 2013 national free delivery care policy.
6.	Asefa et al., (2020)/Ethiopia	1. Mixed methods study 2. Comparative before-and-after evaluation design. 3. To describe the lessons learned in Respectful Maternity Care training and its implementation from the perspectives of service providers’ perceptions and experiences.	1. 64 service providers 2. Service providers (midwives, integrated emergency surgical officers, nurses, general practitioners, and other health officers).	1. Respectful Maternity Care training for service providers. 2. 3 days. 3. No. 4. Pre – and- post surveys and post intervention focus groups. 5. Pre- and -post Respectful Maternity Care intervention survey and focus groups 2 months after the intervention. 6. McNemar’s test for quantitative data and hybrid thematic analysis for qualitative data. 7. The quantitative study assessed participants’ experience of mistreatment of women in their facilities and compared participants’ perceptions of Respectful Maternity Care before and after the intervention. Eight questions representing different categories of mistreatment (non-consented care; lack of information, privacy and confidentiality; physical abuse; verbal abuse; refusal of preference; neglect, and discrimination) were used to assess whether participants had witnessed mistreatment of women in their hospital. The qualitative study explored participants’ perceptions of Respectful Maternity Care and the challenges encountered when implementing it.	The training improved the participants’ awareness of the rights of women during childbirth and their perceptions and attitudes about Respectful Maternity Care were positively influenced. However, participants believed that the Respectful Maternity Care training did not address providers’ rights. Structural and systemic issues were the main challenges providers reported when trying to implement Respectful Maternity Care in their contexts.
7.	Oosthuizen et al., (2020)/South Africa	1. Quantitative study. 2. Comparative before-and-after evaluation design with a control group. 3. To determine the effect of the ‘CLEVER Maternity Care’ package, a multi-faceted intervention to improve respectful, quality obstetric care.	1. At baseline 653 women and after implementation of CLEVER 679 women. 2. Midwife-led obstetric units.	1. CLEVER Maternity Care package. 2. 9 months. 3. Yes. 4. The baseline and end-line surveys were conducted for both the intervention and non-intervention MOUs using a self-administered survey tool. 5. Pre – and – post intervention measurements. 6. Weighted logistic regressions, odds ratios. 7. Experiences regarding communication, labor, clinical care and respectful care during confinement, clinical care received, and client satisfaction.	There was no significant change in the proportions of responses with regard to patients receiving attention within 15 min of arrival, both the intervention and control group units showed a significant increase in positive responses (odds ratios of 8.4 and 6.1, respectively, and *p* values of 0.0001 and 0.0007). For asking permission before doing an examination, positive communication, respectful treatment, and overall satisfaction, only the intervention group showed a significant positive change (odds ratios ranging from 2.4 to 4.3; *p* ≤ 0.0018), with no significant change for the control group (odds ratios between 1.0 and 1.8; *p* ≥ 0.0736).
8.	Afulani et al., (2018)/Ghana	1. Quantitative study. 2. Comparative before-and-after evaluation design. 3. To evaluate the effect of integrated simulation-based training on the provision of respectful maternity care.	1. 215 women at baseline and 318 women at endline. 2. Service providers (midwives, medical doctors, anesthetists, nurses).	1. Integrated simulation-based training on the provision of respectful maternity care. 2. 2-day training and four 3-h refresher training once a month. 3. No. 4. Exit interviews with recently delivered women pre-and-post intervention. 5. Pre- and-post intervention. 6. Descriptive statistics, chi-squared test, two-sample *t*-test, ordinary least-squares regression. 7. Experiences of care using the person-centered maternity care scale.	Compared to the baseline, women in the endline reported more respectful care. The average person-centered maternity care score increased from 50 at baseline to 72 at endline, a relative increase of 43%. Scores on the subscales also increased between baseline and endline: 15% increase for dignity and respect, 87% increase for communication and autonomy, and 55% increase for supportive care. These differences remained significant in multivariate analysis controlling for several potential confounders.
9.	Swahnberg et al., (2019)/Sri Lanka	1. Quantitative study. 2. Comparative before-and-after evaluation design 3. To assess the potential of the Forum Play training method to increase staff awareness of obstetric violence and promote taking action to reduce or prevent it.	1. 20 physicians and 30 nurses. 2. Physicians and nurses.	1. Intervention using a participatory theatre technique called Forum Play for service providers. 2. Half-day workshops. 3. No. 4. Pre – and –post intervention surveys. 5. Before intervention and 3–4 months after the intervention. 6. Chi-square and Fisher’s exact test. 7. 4 questions about abuse in health care.	At follow-up, participants more often reported that they had been involved in situations of obstetric violence, indicating new knowledge of the phenomenon and/or an increase in their ability to conceptualize it. The intervention appears promising for improving the abilities of healthcare providers to recognize obstetric violence, the first step in counteracting it.
10.	Misago et al., (2001)/Brazil	1. Mixed methods study. 2. Comparative before-and-after evaluation design. 3. To compare the delivery and childbirth situation before and after the project’s intervention activities.	1. At the baseline 279 interviews and observations were carried out, whereas at the endline 348 interviews and observations were conducted. 2. Health professionals.	1. Maternal and Child Health Improvement Project. This project focused on ‘humanization of childbirth,’ with training-based intervention activities. 2. Around 3 years. 3. No. 4. Pre – and – post project surveys using Rapid Anthropological Assessment Procedure. 5. Before and after the project. 6. NR. 7. Ten different instruments were used: community form F-1 was applied to a small number of community leaders. Mothers were interviewed to identify health priorities, and then to collect accounts of childbirth using F-2. Men were interviewed on their health priorities and childbirth-related experiences, using F-3. Form 4 was administered to a convenient sample of birth attendants, either hospital-trained or traditional, who were identified through F-1. F-5 was applied to physicians and other health professionals from the municipalities. F-6 and F-8 were used by specially trained observers in maternity wards in the municipalities to collect information on birthing assistance and practices. An inventory of the same hospitals was recorded using F-7. Interviews about maternal and neonatal mortality, using F-9 and F-10, were conducted with every available case that occurred in 1995 and 1996 for the 1997 survey, and 1989 and 1999 for the 2000 survey.	Changes from ‘a culture of dehumanization of childbirth’ to ‘childbirth as a transformative experience’ were observed.
11.	Umbeli et al., (2014)/Sudan	1. Quantitative study 2. Comparative before-and-after evaluation design. 3. To assess the impact of health care providers training on patient- provider’s communication during childbirth.	1. 225 health care providers (120 pre – and 105 post) and 4,469 women (2,000 pre – and 2,469 post). 2. Registrants, house officers, midwives, and data collectors.	1. Training for improving patient-provider’s communication. 2. NR. 3. No. 4. Pre – and – post intervention interviews. 5. NR. 6. Chi-square test. 7. Information given to patients on admission to labor ward, permission and consent for procedures, postnatal follow-up, and patient’s satisfaction.	The study showed that training health care providers on communication skills has an effective improvement in health care providers’ knowledge and practice toward communication with patients in many areas during labor, resulting in good patient satisfaction. However, improvement in communication skills needs sustained in-service training.
12.	Infanti et al., (2020)/Sri Lanka	1. Qualitative study 2. Descriptive qualitative research design. 3. To assess the acceptability and feasibility of the Forum play method to tackle abuse in health care.	1. 23 intervention participants (10 physicians and 13 nurses). 2. Physicians, nurses.	1. Forum Play training intervention to increase awareness of abuse in health care and promote taking action to reduce or prevent it. 2. Half-day workshop. 3. NA. 4. Focus groups. 5. After the intervention. 6. Content analysis. 7. Acceptability and feasibility of the intervention.	The intervention method stimulated dialogue and critical reflection and increased participants’ awareness of the everyday nature of abuses experienced by patients. Participants appreciated the participatory format of Forum Play, which allowed them to re-enact scenarios they had experienced and rehearse realistic actions to improve patient care in these situations. Structural factors were reported as limitations to the effectiveness of the intervention, including under-developed systems for protecting patient rights and reporting health provider abuses. Nonetheless, the study indicates the acceptability and feasibility of a theatre-based training intervention for reducing the mistreatment of patients by healthcare providers in Sri Lanka.
13.	Dzomeku et al., (2021)/Ghana	1. Qualitative research. 2. Descriptive qualitative research design. 3. To explore midwives’ experiences of applying Respectful maternity care knowledge in their professional practice after participating in a four-day training program.	1. 14 intervention participants. 2. Midwives.	1. Respectful Maternity Care modules (respect and dignity in childbirth, communication during childbearing women’s care, focused antenatal care, effective, alternative birthing positions). 2. 4-day program. 3. NA. 4. In-depth interviews. 5. After the intervention. 6. NR 7. Experiences of applying the acquired Respectful maternity care knowledge in daily maternity care practices.	Despite the report of some Respectful maternity care implementation challenges, the midwives noted that the 4-day Respectful maternity care training has had a positive impact on their maternity caregiving practice in the hospital. Policies and programs aimed at addressing the issue of disrespect and abusive practices during maternity care should advocate and include the building of facilities that support alternative birthing positions and the privacy of childbearing women during childbirth.
14.	Zbikowski et al., (2020)/Sweden	1. Quantitative study. 2. Comparative before-and-after evaluation design 3. To explore to what extent the intervention using Forum play increases the staff’s awareness of abuse in health care and their ability to take action against it.	1. 92 staff members at baseline, 85 staff members during an intervention, 79 staff members after the intervention, and 78 staff members one-year follow-up. 2. Women’s clinic staff (physicians, midwives, auxiliary nurses, secretaries).	1. The improvised role-play method Forum Play (FP), based on techniques developed by Boal with health care staff. 2. 16 workshops for 3–3.5 h in a year. 3. No. 4. Self-reported questionnaires. 5. Before, during, and after intervention, and one-year follow-up. 6. Wilcoxon signed rank test and McNemar’s test. 7. The number of reported occasions of abuse in health care and Forum Play participants’ ability to act in those situations.	An increase in the participants’ self-reported ability to act in abuse in healthcare-related situations. However, no change is observed in the number of reported occasions of abuse in health care between baseline and 1 year after the intervention. Health care staff’s participation in workshops using improvised role-play can increase staff’s perceived ability to take action in abuse in health care situations. The voluntary nature of the intervention may have attracted those who were already aware of the topic, and likely explains the unchanged awareness of abuse in health care.
15.	Diniz et al., (2020)/Brazil	1. Mixed methods study. 2. Participatory action research. 3. To develop and implement a Mother Baby-Friendly Hospital Initiative (MBFHI) in an academic maternity hospital in Brazil and evaluate how change could be sustained.	1. NR. 2. Clinicians and maternity hospital managers.	1. Mother Baby-Friendly Hospital Initiative (MBFHI). 2. Nine formal sessions were conducted, with a mean duration of 100 min and many informal meetings over the course of 2 years. 3. NA. 4. Observation, interviews, focus groups, and historical and documentary analysis 5. NR. 6. Thematic analysis. 7. Situation at baseline and changes in the process.	Although challenges remained, positive changes included a friendlier environment, improved patient privacy, and fewer unnecessary procedures. Resources released by these changes allowed us, collaboratively, to track the further implementation and sustainability of change.
16.	Afulani et al., (2020)/Ghana	1. Mixed-methods study. 2. Comparative before-and-after evaluation design 3. To examine the effectiveness of integrated simulation training in emergency obstetric and neonatal care and respectful maternity care on providers’ knowledge and self-efficacy, and to assess providers’ perceptions of the integrated training.	1. 43 providers filled out the self-administered evaluation forms and 17 gave in-depth interviews. 2. Service providers.	1. Integrated simulation training on emergency obstetric and neonatal care and respectful maternity care. 2. 2-day training and four 3-h refresher training once a month. 3. No. 4. Self-administered evaluation forms, in-depth interviews 5. Pre- and post-intervention self-administered evaluation, 6 months follow-up. Interviews one week after the training. 6. Descriptive quantitative analysis and framework qualitative analysis. 7. Knowledge and confidence in emergency obstetric and neonatal care skills and respectful maternity care. Perceptions of the training.	Provider knowledge increased from an average of 61.6% on the pre-test to 74.5% on the post-test. Self-efficacy also increased from an average of 5.8/10 at the pre-test to 9.2/10 at the post-test. Process evaluation data showed that providers valued the training. Over 95% of participants agreed that the training was useful to them and that they would use the tools learned in the training in their practice. Overall, providers had positive perceptions of the training. They noted improvements in their knowledge and confidence to manage obstetric and neonatal emergencies, as well as in patient-provider communication and teamwork. Many listed respectful maternity care elements as what was most impactful to them from the training.

NR, not reported; NA, not applicable.

#### Participants and sample

All the included studies involved an intervention at a medical facility, comprising a total of 16 studies. The sampling process encompassed both women and/or medical staff. Specifically, women who had given birth were recruited after their discharge (30). Women between the ages of 15 and 45 who had delivered within 24–48 h at a participating facility were included (19). Women who had recently given birth at any of the 10 midwife-led obstetric units (MOUs) and subsequently visited primary healthcare facilities for a postnatal follow-up appointment within 3 days to 6 weeks post-delivery were included (35). One study selected a sample from 30 births that had been monitored using the Federal Ministry of Health of Ethiopia (FMoH) adopted Safe Childbirth Checklist (SCC) in the previous month (31). The majority of the studies focused on healthcare providers (19, 30–44). The length of intervention varied from half a day (37, 42) to around 3 years (39). One study measured the long-term impacts for 10–11 months following training with total data collected over a period of 27 months (31), while another study conducted workshops on abuse for healthcare staff (AHC) for 13 months (40). Additionally, one study assessed outcomes 10 months after the intervention (30).

Qualitative data in mixed methods and qualitative studies was derived from various empirical tools. These included direct observation of interactions between clients and providers (33), interviews with women after discharge to identify potential mechanisms of change (32), interviews conducted with women 3–6 h after delivery in one study [baseline *N* = 2000, (33)], community follow-up interviews with women conducted 4–6 weeks after delivery [baseline *N* = 70, (33)], structured focus group discussions (FGDs) with midwifery, gynecologists (32), FGDs with health professionals who were participants in RMC training (34, 42); in-depth interviews with participants of the RMC training (43), FGDs with health professionals with experiences supporting and listening to pregnant women during community engagement activities (31).

### Factors influencing obstetric violence

The factors influencing obstetric violence can be categorized into technical and interpersonal aspects. Most acts of disrespect and abuse occurred within the interactions between healthcare providers and women. Interpersonal violence and abuse were found to be associated with several key issues addressed in the interventions. Firstly, non-confidential care was a prominent concern addressed in 11 studies (19, 30–34, 36, 39, 42–44). Secondly, the issue of non-dignified care was highlighted in seven interventions (19, 30, 32, 33, 36, 41, 43). Thirdly, non-consented care was addressed in seven studies (19, 30, 32–35, 44). Additionally, questions relating to feelings of neglect, humiliation, and disrespect (19, 32, 34, 35), verbal abuse (19, 34, 35, 41), detention (19, 32), abandonment (19, 32, 34), denial of the right to free care or denial of care (32, 44), and physical abuse (19, 30, 32, 34) were also examined in multiple studies. Table 3 shows the synthesis of the results.

**Table 3:** Synthesis of the factors whose association with OV was observed in the interventions.

Interactional level	Descriptive factors	Negative factors before intervention	Positive factors after intervention
	Non-dignified care	√ √ √ √ √ √ √ √ ^19,30–33,42–44^	
	Non-consented care	√ √ v √√ √ √ √ ^19,30,32–35,44^	
	Feelings of neglect, humiliation	√ √ √ √ √ ^19,30,32,34,35^	
	Verbal abuse	√ √ √ √ ^19,34,35,41^	
	Abandonment	√ √ √ ^19,32,34^	
	Lack of communication skills	√ √ √ ^36,38,39^	
	Providers that do not introduce themselves	√ ^36^	
	Providers that do not ask permission for examinations	√ ^36^	
	Normalization of mistreatment	√ ^34^	
	Uterine contractions or other signs rarely monitored	√ √ √ ^19,32,39^	
	Detention – Women restricted from movements	√ √ ^19,32^	
	Unnecessary procedures, such as shaving of pubic hair	√ ^39^	
	Patients’ rights underdeveloped	√ ^42^	
	Denial of right to free care	√ √ ^32,44^	
	Punitive action against “uncooperative women”	√ ^34^	
	Dignity and respect – a decrease of:		√ ^19,30,32,33,35,36,38^
	Non-consented care, examination		√ √ ^32,38^
	Physical abuse		√ √ √ ^19,30,32^
	Detention		√ ^19^
	Increased access to care		√ ^32^
	Inclusive and collaborative care		√ √ ^42,44^
	Improved patient provider communication		√ √ √ √ √ √ √ √ √ ^31,33,35,36,38–40,42,43^
	Create space of open discussion		√ √ √ ^36,37,43^
	The right to be informed		√ √ √ √ √ √ ^19,33,35,36,38,43^
	Knowledge of ethics, conduct and patients’ rights		√ √ √ ^33,34,42^
	Birth position choice		√ ^34^
	Autonomy, trust and safety		√ √ ^36,43^
	Greater capacity to empathize		√ √ √ √ √ ^19,33,36,40,42^
	Person-centered maternity care		√ ^36^
	Sense of collective experiencing or understanding		√ √ ^37,42^
Organizational level	Non-confidential care	√ √ √ √ √ √ √ √ √ √ √ ^19,30–34,36,39,42–44^	
	Lack of organization of services	√ ^39^	
	Delivery and labor rooms noisy, unventilated and hot	√ ^39^	
	Lack of institutional support to report abuse	√ ^42^	
	Allowing birth companion		√ √ √ √ √ ^31,33,34,36,39^
	Improving facility conditions		√ ^32^
	Confidentiality and privacy		√ √ √ √ √ ^30–32,39,43^
	Creation of separate rooms		√ √ ^30,44^
	Use of curtains of service		√ √ ^30,44^
	Use drapes or cover for intimate examination		√ ^32^
	Quiet and ventilated rooms, relaxing music		√ ^39^
	Freedom to move		√ √ √ ^39,42,44^
	Possibility of adapting birthing positions		√ √ √ ^36,39,43^
	Tour wards and cultural adaptation		√ ^31^
	Provider job satisfaction		√ ^33^
	Commitment and institutional leadership		√ ^42^
	Mentoring and motivation of providers’ performance		√ √ ^35,38 32^

√ Represents the number of studies reporting the respective association or observation.

#### Obstetric violence interventions

In the 16 studies, a total of 10 different types of interventions were identified, ranging from collaborative building curriculum to childbirth checklists. The interventions included a variety of approaches, such as the implementation of client service charters, employing training programs for healthcare providers, using simulation-based team training, changing laboratory methodology, promoting humanization of childbirth initiatives, conducting forum play workshops, and providing communication skills training. Due to the diversity of studies, some research presents outcomes derived from several different approaches.

#### Respectful Maternity Care (RMC)

Four studies utilized an approach based on Respectful Maternity Care (RMC), combined with patient-centered care, client-provider interaction, and family participation, to address abuse and disrespect (32–34, 43). This approach recognizes that providing compassionate and respectful maternity care during pregnancy is crucial for improving the quality of maternal health services and reducing maternal mortality and morbidity. The World Health Organization (45) defines RMC as “the care organized for and provided to all women in a manner that maintains their dignity, privacy, and confidentiality, ensures freedom from harm and mistreatment, and enables informed choice and continuous support during labor and childbirth.”

#### The safe childbirth checklist

The Safe Childbirth Checklist collected data on privacy and the availability of a birth companion during labor and childbirth over a 27-month period from 17 health centers and three hospitals (31). This intervention was based on the Ethiopian government’s decision to motivate health professionals to adopt a Compassionate, Respectful, and Caring (CRC) approach. The goal was to institutionalize RMC by integrating, training, and empowering health professionals through life-testimonial video-based training that included participatory discussions, reflections, and local solutions to enhance RMC. Successful ideas for improving women’s care experiences were shared during staff meetings. This intervention involved the collaboration of all facilities at the district level from 12 to 15 months.

#### The PRONTO training kit

Afulani et al. (36, 41) implemented a collaborative approach using the PRONTO training kit. This low-tech, highly realistic simulation and team training, accompanied by facilitated debriefing, aimed to improve the identification and management of obstetric and neonatal emergencies, as well as team functioning. The PRONTO training kit included a hybrid birth simulator called the PartoPantsTM, which consisted of modified surgical scrubs with anatomical landmarks necessary for delivery. The intervention focused on enhancing the quality of care by emphasizing clinical knowledge and skills, teamwork and communication, and RMC. While RMC was emphasized throughout the PRONTO training, there were no explicit modules solely dedicated to RMC. The curriculum included simulation scenarios, debriefing guides, knowledge reviews, and interactive teamwork and communication activities.

#### Mother baby-friendly hospital initiative (MBFBF) 10 criteria – the change laboratory methodology

The interventions focused on implementing change: the Change Laboratory (CL) methodology (44). The research initially started in late 2016, and the CL principles were adopted 9 months later, following adjustments to better incorporate the conceptual framework. The CL methodology aimed to address 10 key challenges related to unfriendly childbirth care in hospitals. These challenges include issues such as freedom of movement, eating and drinking during labor and birth, respect for privacy, right to companionship, use of evidence-based care and prevention of inappropriate interventions, freedom from discrimination, freedom from physical and emotional abuse, access to appropriate pain relief, affordability or free care, cultural sensitivity, and ensuring appropriate care for the newborn, including facilitating skin-to-skin mother-baby contact.

#### The Staha project theory of change and open birth days

The Staha Project Theory of Change (30, 33), was developed through an iterative participatory process involving local community and health systems stakeholders. It consisted of two main components. Firstly, a client service charter was created to establish consensus on norms and standards that promote mutual respect and respectful care. The charter was adapted by the two groups and then revised based on feedback from another group of 70 stakeholders (86% provided feedback). Subsequently, the charter was disseminated to communities and displayed in health facilities within the intervention district. Secondly, a maternity quality improvement process was implemented to activate the content of the charter. Planned interventions included the implementation of the Health Workers for Change curriculum by Ratcliff et al. (33) and the Open Birth Days (OBD) intervention. These interventions aimed to increase patient knowledge about their rights, and birth preparedness, improve patient-provider and provider-administrator communication, and enhance women’s experiences and provider attitudes.

#### The Heshima’s project

Launched in 2011 in Kenya, the Heshima project included complementary interventions at community-level, facility-level, and policy-level (19). This project also performed quantitative and qualitative assessments to test associations between the implementation of D&A activities and trends in quality of care at intervention facilities. This project included an iterative three-tiered set of interventions of a process of learning-by-doing throughout its design, development and assessment, with the objective of identifying low-cost and feasible policy, facility and community interventions. Facility interventions, which began in six facilities for 20 months, were replicated and refined in seven facilities beginning in November 2012 and continued for 14 months. At the facility level, intervention activities included: training in promoting RMC, quality improvement teams, caring for carers, D&A monitoring, mentorship, and maternity open days. At the community level, interventions included workshops in the community, mediation/dispute resolution and counseling community members.

#### The CLEVER maternity care and humanization of childbirth

CLEVER Maternity Care stands for Clinical Care and obstetric triage and is about eliminating barriers to care, verifying and monitoring, reflexive and respectful care, and emergency obstetric simulation training (EOST). Oosthuizen and colleagues measured perinatal morbidity and mortality as proxies for quality obstetric care (35). The intervention was implemented in three phases: creating awareness, implementing core activities for behavioral change, and conducting follow-up on the baseline survey.

Humanization of Childbirth involves integrating the Japanese midwifery concept of a “safe and satisfied birthing experience.” This approach aims to empower women, promote active participation and decision making and ensure equality. It utilizes a decentralized system of birth and seeks to be evidence-based as well as financially feasible. A self-controlled experimental study before-and-after design was used to compare the delivery and childbirth situation in five municipalities in a Brazilian state (39). Data was collected at two time points: 1997 and 2000. The participatory intervention firstly consisted of several training activities, including seminars, workshops, in-service training, and training for trainers, and secondly by grouting the learning on participants’ conscious taking.

### Forum play workshops

These trainings are based on the pedagogical work of Brazilian stage director, Augusto Boal (Theater of the Oppressed and Forum Theatre) and Paulo Freire, as well as on problem-posing dialogue and empowerment. These methods have been used over several decades on curricula and training development to promote participation, personal and collective reflection and transformative actions in oppressive or ethically complex contexts. The intervention consisted of providing workshops and training to strengthen participants’ abilities to recognize abuse in healthcare and to improve readiness to act in D&A situations (37, 40, 42).

#### Training on communication skills

This is a quasi-experimental study to improve patient providers’ communication in the labor ward at Omdurman Maternity Hospital, Sudan (38). A situation analysis prior to training, evaluating the existing communication skills of providers and patients’ satisfaction was carried out. A 10% sample of hospital deliveries and all healthcare providers (HCPs) were included in the study before and after the training. The study examined socio-demographic characteristics and evaluated the information given to patients upon admission to the labor ward, as well as the process for obtaining permission and consent for procedures, post-natal follow-up, and patients’ satisfaction before and after training.

### Assessment of interventions: main outcomes

From our findings, the greater number of interventions reviewed demonstrated positive outcomes in reducing obstetric violence with only a few exceptions recording no changes post intervention. In the following section, we describe the outcomes of the various studies reviewed in this study.

#### Enhanced patient-centered communication skills

These interventions focused on improving communication between healthcare providers and women during childbirth. This included creating space for open discussions, encouraging questions, improving patient-provider communication, and guaranteeing women’s right to be informed. Some interventions even included activities such as organizing tours of the birthing ward and allowing cultural celebrations (31). These interventions resulted in increased empowerment, trust, empathy, emotional support, and a more inclusive and collaborative care environment, subsequently leading to an overall reduction in obstetric violence. The enhancement of patient-centered communication skills emerged as a key factor in achieving this reduction. Two specific communication skills were found to have a strong influence. Firstly, creating space for open discussion and encouraging questions empowered women throughout the entire process of childbirth. Several interventions underscored the significance of encouraging communication, recognizing its pivotal role in the childbirth process (31, 35, 36, 39, 42, 43). Secondly, improving patient-provider communication and the right to be informed was identified as crucial in reducing obstetric violence (19, 33, 35, 36, 38, 43). Furthermore, Ratcliffe and colleagues (33) noted a significant improvement in provider knowledge following RMC training. Specifically, there was a 5.4% increase in understanding their code of conduct, ethics, and patient rights. Additionally, a 6.8% increase was observed in their awareness that disrespect and abuse represent a global problem. Creating a trusting and safe environment, providing more empathy and emotional support, and promoting inclusivity, collaboration, and freedom of movement (such as allowing drinking or eating) were also noted as positive factors in reducing obstetric violence (19, 36, 42, 44). Specifically, Afulani et al. (36) observed an 87% increase in communication and autonomy. Indeed, they observed higher changes in the domain of communication and autonomy, with the score nearly doubling. Additionally, Mengistu et al. (31) observed that testimonial videos proved helpful for providers to understand their care from the perspective of their patients. At the same time, quality improvement training and coaching facilitated reflection on potential underlying causes of mistreatment and the development of effective solutions.

#### Increased birth companion and privacy

Interventions aimed at increasing confidentiality and privacy in childbirth settings implemented various strategies. These included organizational changes such as creating private or separate rooms for admissions, antenatal care, family planning, and postnatal care, which were previously provided in a single room (30, 31). Additionally, the use of curtains for delivery, examinations, and between beds was employed to enhance privacy (30, 44). A study by Miret et al. (32) assessed non-confidential care and reported a 54.8% reduction in events of non-confidentiality after the intervention, attributing it to the use of drapes or covering intimate parts and ensuring private health information was not discussed in a way that others could hear. Another intervention introduced changes where mothers no longer had to share rooms, allowing for greater privacy, especially when empty rooms were available (44). Some interventions also explored the possibility of having a birth companion in the delivery room, even when multiple women were delivering simultaneously. The findings indicated a positive shift, with a higher percentage of women having a birth companion at the endline compared to the baseline. Specifically, Afulani et al. (36) observed a noteworthy decrease in the proportion of women who reported never being allowed to have a companion during labor, declining from 32% at baseline to 10% at the endline. In the context of open labor wards, the introduction of the option to change birthing positions for enhanced privacy was implemented, as highlighted in the study by Dzomeku et al. (43). The findings suggest that a dedicated refresher training course focusing on provider attitudes, knowledge, and best practices holds the potential for a positive impact on the maternity care environment. Additionally, Ratcliffe et al.’s study (33) observed a significant improvement in patient knowledge following pre and post-tests administered during Open Birth Days sessions. The results demonstrated a noteworthy increase in awareness of various rights during labor and delivery, including the right to consent (rising from 30.1 to 57.8%), the right to be free from physical abuse (increasing from 79.5 to 86.9%), and the right to privacy (climbing from 68.2 to 81.9%) (33).

Several interventions focused on person-centered maternity care and observed positive changes. A study (36) reported significant improvements in person-centered maternity care features after the intervention, including increased introduction of providers, calling patients by their names, explaining the purpose of examinations, procedures, and medications, and obtaining permission. Overall, the average score for person-centered maternity care increased from approximately 43–72%, indicating a relative increase in dignity and respect, communication and autonomy, and supportive care (36). In the study conducted by Swanhnberg (37), participants reported increased involvement in cases of abuse and a higher rate of acting in support of patients after a workshop intervention. The intervention facilitated open discussion, increased awareness of abuse in healthcare, and addressed factors such as stress and lack of knowledge on how to respond to mistreatment. The workshop format, which included the physical use of bodies for learning and facilitated expression of emotions, was highly effective, according to participant feedback. Another study by Zbikowski et al. (40) found that participation in an educational workshop program improved self-reported ability to act according to moral beliefs in risk situations of abuse and enhanced empathy and communication skills among healthcare professionals.

The oldest study included in the report, conducted in 2001 by Misago et al. (39), highlighted several inadequate practices during childbirth, such as noisy and unventilated delivery rooms lacking privacy, restrictions on movement, unnecessary procedures, and unattended women delivering in labor rooms. However, positive changes were observed in hospitals that received “humanization of childbirth” training, including increased numbers of deliveries, frequent use of birth companions, and encouragement of women to bring a family member or significant other. Indeed, Misago and colleagues noted a significant change over time. In 1997, women in labor were frequently unattended, whereas by 2001, there was a noticeable shift, with women consistently accompanied by someone during labor. Only one out of 12 direct delivery observations identified an unaccompanied woman, highlighting a marked improvement in the presence of support during the childbirth process.

Umbeli et al. (38) reported that training in communication skills led to increased support and respect from providers according to patient perceptions. Providers also reported improved knowledge of various aspects of care after the training, informing women about the birth process more often. For example, improvements were seen in informing women on such aspects as fetal condition (from 65.8 to 96.2%), expected duration of birth (from 22.5 to 81.9%), and examinations/procedures (from 20.0 to 81.0%). In the study conducted by Mengistu et al. (31), significant improvements were observed in the percentage of births that received two important elements of respectful maternal care: privacy and birth companionship. Specifically, one district showed a notable increase, with short and long-term regression coefficients of 18 and 27%, respectively, indicating a positive trend over time. However, the results were mixed in the other two districts included in the study, suggesting that the interventions may have had varying effects in different settings.

#### Decrease of situations of disrespect and abuse

The interventions led to a decrease in the overall prevalence of disrespect and abuse, particularly neglect and physical abuse. Furthermore, the interventions significantly improved the perception of respectful care, with participants reporting excellent or very good ratings for the respect shown by providers and the overall quality of care for delivery.

In the study conducted by Kujawski et al. (30), the intervention showed a significant decrease in the percentage of women who experienced abuse and disrespect during childbirth. The results indicated a 3.39% decrease (*p* < 0.0001) in the overall prevalence of abuse and disrespect. When adjusting for covariates, the intervention was associated with 66% reduced odds (95% CI: 0.21–0.58, *p* < 0.0001) of women experiencing disrespect and abuse. Furthermore, women in the intervention facility were significantly less likely to report instances of neglect (OR: 0.36, 95% CI: 0.19–0.71, *p* = 0.045) and physical abuse (OR: 0.22, 95% CI: 0.05–0.97, *p* = 0.003) when compared to the control facility, after adjusting for all variables in the conceptual model (30). Additionally, the intervention was associated with an increased likelihood of pregnant women rating the respect shown by providers during their facility stay for delivery as excellent or very good (RR: 3.44, 95% CI: 2.45–4.84, *p* < 0.0001), as well as rating the overall quality of care for delivery as excellent or very good (RR: 6.19, 95% CI: 4.29–8.94, *p* < 0.0001).

Afulani et al. (36) observed similar trends with an increase in dignity and respect (15%), and a 55% increase in supportive care after RMC training. Additionally, as reported by Ratcliff et al. (33), reporting situations of D&A increased after intervention, going from no participants reporting these situations at baseline to 10% of women who attended the OBD (Open Birth Days) reporting feeling disrespected during labor and formally filed a complaint. A study (32) revealed that D&A decreased from 71.8% at baseline to 15% at endline with a change of 55% (mean difference: 0.56, 95% CI: 0.55–0.57). Similarly, Abuya and colleagues (19) observed that D&A decreased from 20 to 13% (*p* < 0.0004) and among four of the six typologies of D&A it was observed a decrease of 40–50% and an overall D&A decrease of 7% reported by postnatal women after their discharge from maternity units. Nonetheless, the frequency of typologies varied considerably in both the interviews and observations. Rates of verbal abuse, for instance, were several times higher than rates of physical abuse, in both interviews and observations. Therefore, some D&A typologies declined more than others, with the greatest decline in detention and physical abuse.

Indeed, certain studies only showed modest improvements in dimensions related to D&A. Asefa et al. (34) observed a marginal decrease in the belief that it is sometimes necessary for providers to yell at a woman during labor from 21.9% pre-test to 20.3% post-test, *p* = 1.00. Also, Afulani et al. (36) also observed the smallest changes in dignity and respect, but that might be explained by the relatively high scores at baseline. Zbikowski et al. (40) found that participation in an educational workshop program improved the self-reported ability to recognize abuse and act in situations of abuse by healthcare professionals. However, no change was observed in the number of reported occasions of abuse in healthcare between baseline and 1 year after the intervention (40). These findings suggest a notable enhancement in the perception of respectful care and the overall quality of care among women at the intervention facility when compared to the comparison facility with only one exception (40).

## Discussion

This scoping review comprehensively synthesized existing literature on interventions aimed at reducing obstetric violence and assessed their effectiveness in promoting respectful maternity care during childbirth. In general, research on obstetric violence interventions is relatively new. Although obstetric violence is a global phenomenon with high prevalence cutting across cultures (14–22), the majority of the studies focusing on interventions were conducted in low- and middle-income countries (LMICs), particularly African Countries. While this expressly reveals Africa’s contribution to knowledge production on obstetric violence, it also points to the rigorous efforts being made to address the high maternal mortality rate on the continent. Sub-Saharan Africa currently accounts for two-thirds of the global Maternal Mortality Ratio (MMR) due to the low utilization of Skilled Birth Attendants (SBA) and poor obstetric care, making research on obstetric violence interventions germane (46, 47). In this review, most of the studies involved interventions at a health facility and the sample groups included both women and medical staff, with the majority focusing on healthcare providers. and were published over a range of 20 years, between 2001 and 2021.

Overall, the interventions demonstrated positive outcomes in reducing obstetric violence and enhancing the childbirth experience for women, particularly with integrated provider training contributing to improved childbirth experiences in resource-limited settings. Based on the results of this scoping review, factors influencing obstetric violence are mostly technical and interpersonal aspects. Non-confidential care, non-dignified care, non-consented care, feelings of neglect and disrespect, verbal abuse, detention, abandonment, and denial of the right to free care are the main issues addressed in various intervention programs.

Effective communication emerged as a crucial factor, highlighting the significance of open discussions, the right to be informed, and the promotion of women’s independence during childbirth. This emphasis on communication underscores the critical role of healthcare professionals’ ability to communicate with women both during and after childbirth, as highlighted in previous studies (48, 49). The identified lack of interpersonal communication skills and attitudes is recognized as a significant weakness in obstetric training (50). Established guidelines for enhancing the quality of obstetric healthcare in countries such as Canada, position communication as a central resource (51). Additionally, within the healthcare context, the element of self-presentation to patients is acknowledged as a key communication aspect for establishing a supportive relationship (52). Furthermore, previous research on obstetric violence has revealed that healthcare professionals often hold misconceptions about this issue. A study (3) conducted in Ghana found that a significant number of healthcare professionals do not perceive acts of mistreatment as abusive. Instead, they view such actions as a form of assistance or ‘help’ beneficial to the newborn, justifying their behavior. Similarly, in India, wrong perspectives on obstetric violence were identified as a major driver of these abuses within healthcare facilities (53). Hence, there is a crucial need for studies to specifically target the healthcare team, addressing and rectifying these misconceptions. Regarding the characteristics of the interventions in our scoping review, we identified a total of 10 different types of interventions across the 16 studies. These interventions assessed several strategies and approaches aimed at promoting respectful and patient-centered maternity care. When compared to pre-intervention, there was a significant improvement in patients’ understanding of the labor process in post-intervention. Higher levels of empathy for women and improved interpersonal relationships were reported by the providers. For example, in the study by Ratcliffe et al. (33), 98.2% of participants said that participation in the intervention improved the communication between patients and providers, and there was a 10.8% increase in providers who agreed that they were able to empathize with their patients. Enhancements in communication were observed and confirmed through direct assessments, as noted by both patients and staff. During childbirth, women reported feeling more in control and assured. Along with evaluations of improved happiness and perceived care quality, provider job satisfaction significantly rose relative to the baseline.

Unfortunately, studies are showing that obstetric violence is still widespread. For example, a study (54) revealed that, regardless of the quality of a healthcare system or a country’s economic well-being, almost two-thirds of the countries across the Eastern Mediterranean Region exhibited six out of seven types of disrespect and abuse during childbirth. Women faced various forms of mistreatment during labor, with physical abuse (particularly the excessive use of routine interventions) and non-dignified care (embedded in patriarchal socio-cultural norms) being most prevalent. In addition, numerous studies emphasize the alarming global spread of abusive and disrespectful practices toward women during childbirth (22, 55). Nevertheless, our scoping review’s findings offer a promising perspective, suggesting that interventions aiming at reducing obstetric violence show success and provide hope for the delivery of respectful care.

When considering the main outcomes of the interventions examined in our study, distinct themes emerge, highlighting the pivotal role of improved patient-centered communication skills and increased use of birth companionship and privacy, all of which contributed to a reduction in situations of abuse and disrespect. Primarily, the interventions underscored the significant refining of patient-centered communication skills as a critical factor in reducing obstetric violence. The establishment of a conducive space for open discussion, combined with the encouragement of questions and the right to be informed, emerged as the first two crucial skills in this context. These skills not only fostered women’s autonomy during childbirth but also significantly enhanced communication between patients and providers. Recognizing the importance of good communication between patients and healthcare providers during childbirth aligns with existing literature emphasizing its critical role in childbirth experiences (46, 47). Furthermore, our study’s findings regarding the positive impact of birth companions are consistent with (56) research in Palestine, revealing a 75% reduction in obstetric violence likelihood among women with birth companions. Additional helpful elements in reducing obstetric violence were building trust and a safe environment, offering more empathy and emotional support, and encouraging inclusivity, collaboration, and freedom of movement (such as allowing drinking or eating).

Again, interventions targeted at ensuring privacy demonstrated notable success in diminishing situations of non-confidential care. These measures include the establishment of separate rooms for admissions, antenatal care, family planning, and postnatal care, using curtains for delivery, vaginal examinations, and between beds, and allowing for changes in birthing positions. Studies demonstrate that obstetric violence often involves privacy breaches (7), categorized as common practices such as gross violations of privacy (26), health system incompetence, and lack of physical privacy in health centers for health examinations (24, 57, 58). Castro and Savage (59) also classify the general lack of privacy, the placement of multiple patients in a single hospital bed, insufficient resources for comfort, and the restriction of visitors or family members as typologies of obstetric violence. Considering these perspectives, our scoping review underscores the pivotal role of privacy maintenance as a key outcome of effective interventions. Consequently, our findings highlight the significance of structural investments and raising awareness among healthcare staff about ensuring privacy as primary factors in interventions aimed at reducing obstetric violence. Also, the interventions resulted in a reduction in the overall prevalence of abuse and disrespect, including both physical abuse and neglect. Participants consistently provided high ratings regarding the improvement of respectful care after interventions (30).

Overall, the interventions had a favorable impact on obstetric violence reduction and women’s childbirth experiences. To guarantee respectful and dignified care during childbirth, the results underlined the significance of patient-centered communication, improved privacy, and birth accompaniment, and addressing abuse and disrespect.

## Conclusion

In conclusion, our scoping review examined the multifaceted landscape of obstetric violence interventions, presenting a comprehensive analysis of the available literature. The study identifies key outcomes across 10 interventions, shedding light on their impact on reducing disrespect and abuse during childbirth. Notably, interventions focusing on enhanced patient-centered communication skills, including open discussions and the right to be informed, increased privacy measures, and the involvement of birth companions emerged as pivotal factors in diminishing obstetric violence. Additionally, ensuring privacy through structural investments, such as separate rooms and curtains, significantly decreased instances of non-confidential care. The inclusion of birth companions not only resonated positively with women’s experiences but also demonstrated a substantial reduction in the likelihood of obstetric violence. Overall, the interventions showed positive outcomes. In essence, as obstetric violence continues to be a global health challenge, our study advocates for the continued exploration and implementation of effective interventions to ensure the well-being and rights of childbearing women worldwide.

## Strengths and limitations

This is the first scoping review conducted on obstetric violence interventions in healthcare, providing a comprehensive overview of the intervention studies aimed at obstetric violence reduction. Obstetric violence poses a major threat to women’s reproductive wellbeing, contributing to the global high maternal mortality rate, and our study reveals the potential of interventions to deal with this great challenge. However, the studies included in this review were quite heterogeneous, making it difficult to compare, leading to the decision to undertake a scoping rather than a systematic review. Again, most of the included research focused on specific geographic regions, such as Africa and only studies published in English were included which restricts the generalizability of our findings. More often, health providers (rather than the mothers) were the ones who evaluated changes, gave their opinions, and in some cases, interviewed women about the changes, which could lead to research bias. Furthermore, only two studies included in the review had control groups (30, 35). We recommend that future studies on interventions should evaluate interventions more from women’s experiences, implement controlled-experimental trials with comparison groups, address possible social desirability effects and employ randomization in the sampling. Considering the fact that obstetric violence stems from structural and gender inequality, there is also a critical need for gender-based interventions that address gender stereotyping concerning motherhood, birthing and structural inequalities in health systems.

## Author contributions

AY: Conceptualization, Data curation, Funding acquisition, Methodology, Validation, Writing – original draft, Writing – review & editing. GJ-F: Data curation, Methodology, Validation, Writing – original draft, Writing – review & editing. BK-A: Data curation, Writing – original draft, Writing – review & editing. LA: Formal analysis, Funding acquisition, Validation, Visualization, Writing – original draft, Writing – review & editing.
